# Profound Synergistic Bradycardia: Pharmacokinetic Consequences of Paxlovid-Mediated CYP3A4 and CYP2D6 Inhibition on Ranolazine, Diltiazem, and Metoprolol

**DOI:** 10.7759/cureus.106262

**Published:** 2026-04-01

**Authors:** Simon B Bactawar, Carlos Gaibor, Mohammad Abdul-Waheed

**Affiliations:** 1 Internal Medicine, University of Kentucky, Bowling Green, USA; 2 Cardiology, University of Kentucky, Bowling Green, USA; 3 Interventional Cardiology, The Medical Center at Bowling Green, Bowling Green, USA

**Keywords:** covid19, cytochrome p-450, drug-drug interactions, paxlovid, ritonavir, symptomatic bradycardia

## Abstract

Paxlovid, a codrug of nirmatrelvir/ritonavir, has established itself in the COVID-19 treatment arsenal due to numerous studies documenting clinical efficacy regardless of vaccination status. The ritonavir component, however, is a strong CYP3A4 and strong-to-moderate CYP2D6 inhibitor, reducing the clearance of many cardiovascular drugs. While existing literature documents symptomatic bradycardia from Paxlovid, and single- or dual-drug regimen, a case report of a triple-drug regimen of ranolazine, diltiazem, and metoprolol - utilizing both CYP3A4 and CYP2D6 pathways - is rare. Here we present the case of an 81-year-old female on ranolazine, diltiazem, and metoprolol who developed profound bradycardia (atrial fibrillation with slowed ventricular response) and hypotension due to reduced clearance of these drugs from CYP inhibition.

An 81-year-old female with atrial fibrillation, coronary artery disease, and heart failure was prescribed Paxlovid after testing positive for COVID-19 in the outpatient setting. She subsequently experienced rapid hemodynamic collapse with a heart rate of 41 bpm (from baseline ~66 bpm) and blood pressure of 57/36 mmHg. She was intubated and initiated on multi-drug inotropic support (isoproterenol, dopamine, and epinephrine). Medication reconciliation revealed a home regimen of ranolazine (1000 mg BID), diltiazem (180 mg BID), and metoprolol tartrate (25 mg BID) - all primary CYP 3A4/2D6 substrates. The patient was successfully weaned off of vasopressors and extubated within 48 hours following Paxlovid discontinuation and supportive care. She maintained hemodynamic stability and was discharged the following day.

This case demonstrates that the ritonavir component of Paxlovid can result in the supratherapeutic serum accumulation of ranolazine, diltiazem, and metoprolol through a dual-CYP 3A4 and 2D6 pathway inhibition, leading to a life-threatening bradycardic and hypotensive crisis.

## Introduction

SARS-CoV-2 - a novel and highly contagious beta-coronavirus - is the etiology of the coronavirus disease 2019 (COVID-19), which resulted in a global pandemic [1]. In the United States, COVID-19 became the third leading cause of death in 2020, led by heart disease and cancer [2]. Due to its detrimental effects, rapid vaccination development and treatment options have been introduced, including molnupiravir, remdesivir, and nirmatrelvir-ritonavir (Paxlovid) [3].

Among these, Paxlovid has proven its place in the COVID-19 treatment arsenal due to numerous studies reporting its efficacy in lowering hospitalization rates among patients diagnosed with COVID-19, regardless of vaccination status [4]. The ritonavir component primarily boosts the systemic efficacy of nirmatrelvir (a SARS-CoV-2 3CLpro protease inhibitor), and does so via rapid and strong cytochrome P450 3A4 isoenzyme and moderate-to-strong 2D6 isoenzyme inhibition [5,6]. The U.S. FDA, regarding its prescribing label on ritonavir, has identified multiple cardiovascular medications - such as beta-blockers, calcium channel blockers, and ranolazine - that can be affected by the inhibition of these pharmacokinetic pathways [7].

While SARS-COV-2 can independently precipitate bradyarrythmias and heart-blocks [8], existing literature has demonstrated hemodynamic collapse from Paxlovid itself, and also with its interaction with mono- or dual-cardiovascular drug regimen. Case reports involving a triple-drug cardiovascular regimen with simultaneous engagement of two distinct CYP pathways (3A4 and 2D6) remain exceedingly rare.

Here, we report the case of an 81-year-old female who developed symptomatic bradycardia after taking Paxlovid for a diagnosed COVID-19 infection.

## Case presentation

**Figure 1:**
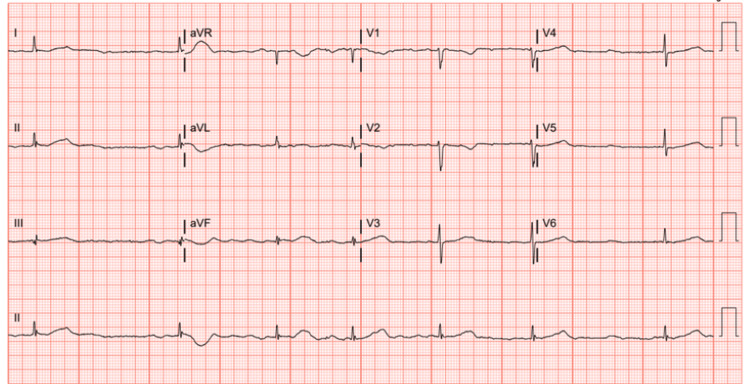
Admission 12-lead electrocardiogram demonstrating atrial fibrillation with a slow ventricular response (SVR), rate of 40 bpm.

**Figure 2:**
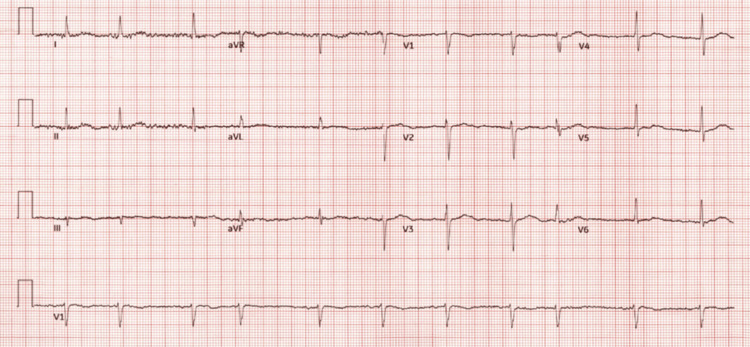
Baseline 12-lead electrocardiogram obtained two months before hospitalization demonstrating atrial fibrillation with a rate-controlled ventricular response (66 bpm).

An 81-year-old female with a history of coronary artery disease, atrial fibrillation, preserved ejection fraction heart failure, and anxiety presented to her primary care physician with complaints of fever, severe sore throat, and body aches. A viral swab assay was positive for COVID-19, and she was prescribed standard Paxlovid therapy (300 mg nirmatrelvir/150 mg ritonavir, twice daily for five days). The patient became progressively confused over the next three days and subsequently became unresponsive. Emergency Medical Services (EMS) was notified with initial vital signs demonstrating profound bradycardia - with a heart rate of 41 bpm (Figure 1) - and hypotension with blood pressure of 57/36 mmHg; baseline electrocardiogram for comparison is shown in Figure 2. Evaluation of oxygen saturation and blood glucose were 92% and 143 mg/dL (reference range 76-106 mg/dL), respectively. En route to the hospital, she received a total of 1.4 L of normal saline fluid resuscitation without hemodynamic improvement. At this time, home diltiazem (180 mg twice daily), ranolazine (1000 mg twice daily), metoprolol tartrate (25 mg twice daily), and Paxlovid were held.

Laboratory analysis of her presenting hematology and metabolic profiles were non-contributary for acute derangements, as outlined in Table 1 - although normocytic anemia and hyperchloremia are present, these were stable baselines for our patient. Her hyperchloremia could have also been exacerbated by normal saline administration. Assay of thyroid function was unremarkable with a thyroid-stimulating hormone (TSH) of 1.330 mIU/L (reference range 0.456-4.680 mIU/L), and free T4 of 1.38 ng/dL (reference range 0.78-2.19 ng/dL). Serum levels of ranolazine, diltiazem, and metoprolol were not obtained during this acute phase of care.

**Table 1:** Laboratory results at initial presentation, with comparison to baseline from prior hospitalization and reference ranges. * Comparison baseline was obtained during a prior hospitalization for acute hypoxic respiratory failure due to entero/rhinovirus six months before the current presentation. Units of measurement used: K/µL: thousands per microliter; M/µL: millions per microliter; g/dL: grams per deciliter; fL: femtoliters; mmol/L: millimoles per liter; mg/dL: milligrams per deciliter

Assay/Test	Patient's Result at Presentation	Patient's Result From Prior Admission*	Reference Range
White Blood Cell Count	6.3 K/µL	7.8 K/µL	4.8-10.8 K/µL
Red Blood Cell Count	2.88 M/µL	3.52 M/µL	4.2-5.4 M/µL
Hemoglobin	8.4 g/dL	8.0 g/dL	12-16 g/dL
Hematocrit	27.4%	29.0%	37-47%
Mean Corpuscular Volume	95 fL	93 fL	81-99 fL
Platelet Count	317 K/µL	349 K/µL	140-440 K/µL
Sodium	139 mmol/L	144 mmol/L	137-145 mmol/L
Potassium	4.6 mmol/L	3.5 mmol/L	3.5-5.1 mmol/L
Chloride	112 mmol/L	113 mmol/L	98-107 mmol/L
Carbon Dioxide/Bicarbonate	23 mmol/L	24 mmol/L	22-30 mmol/L
Blood Urea Nitrogen	11 mg/dL	11 mg/dL	7-17 mg/dL
Creatinine	0.5 mg/dL	0.56 mg/dL	0.52-1.04 mg/dL
Glucose	143 mg/dL	169 mg/dL	74-106 mg/dL
Magnesium	1.9 mg/dL	1.8 mg/dL	1.6-2.3 mg/dL

A dose of intranasal Narcan was administered without success. Atropine 1 mg intravenously was provided with an improvement of heart rate to 63 bpm. Given her state of encephalopathy, the ICU performed intubation for airway protection and started her on isoproterenol (at 2 mcg/min), dopamine (at 5 mcg/min), and epinephrine (at 7.5 mcg/min) drips with improvement of blood pressure to 107/55 mmHg and heart rate to 72 bpm.

Discussion with the daughter revealed that the patient had no additional dose of her home medications and reported no additional change to her regimen except for the newly added Paxlovid.

**Figure 3:**
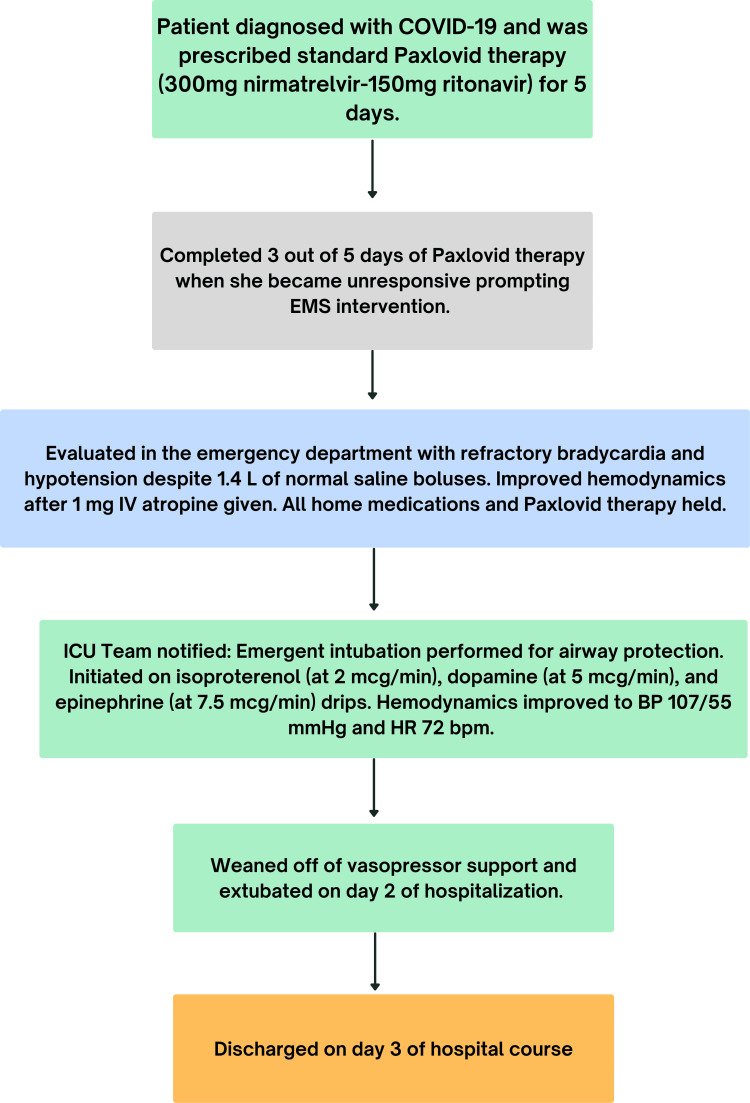
Flowchart showing the chronological summary of the patient's clinical course from diagnosis of COVID-19 to hospital discharge. EMS: Emergency Medical Services; BP: blood pressure; HR: heart rate

During her ICU course, the patient's mental status gradually improved - while mechanically ventilated, she was able to spontaneously open her eyes and display strong symmetrical hand grip. Given this improvement, she was extubated and weaned from all vasopressor support on day 2 of hospitalization. At this point, her complete transthoracic echocardiogram (TTE) revealed a preserved left ventricular ejection fraction of 50-55% without valvular pathology, pericardial effusion, or evidence of inflammatory heart disease. A comprehensive infectious workup, including blood and sputum cultures, was negative. The patient was monitored for an additional day by the hospitalist team and was discharged on hospital day 3 (Figure 3).

## Discussion

COVID-19 remains a frequently encountered etiology in the outpatient setting for patients presenting with viral respiratory symptoms. The approval of oral antiviral agents to avoid future hospitalization has been instituted, with nirmatrelvir-ritonavir (Paxlovid) becoming one of the standard outpatient treatments. With its increased clinical use, the challenges and risks associated with its complex pharmacokinetic profile are now being brought to the clinical forefront [1].

Paxlovid is comprised of two distinct drug components: nirmatrelvir and ritonavir. Nirmatrelvir functions via SARS-CoV-2 3CLpro protease inhibition - preventing the cleavage and maturation of SARS-CoV-2 viral proteins. The ritonavir component functions as a rapid pharmacological booster for nirmatrelvir, increasing its systemic efficacy. Crucially, ritonavir achieves this mechanism of action by strong cytochrome P450 3A4 (CYP3A4) and moderate-to-strong CYP2D6 isoenzyme inhibition activity [1,5,6,8].

As highlighted in the *Journal of the American College of Cardiology* (JACC) review, inhibition of these cytochrome P450 pathways by ritonavir diminishes the primary clearance of several cardiovascular agents. Ranolazine and diltiazem both demonstrate a strong dependence on the CYP3A4 isoenzyme for primary clearance. The suppression of CYP3A4 activity likely allowed serum concentrations of these agents to reach toxic, supratherapeutic levels, precipitating bradycardia and hypotension [9,10].

Notably, a concomitant bradycardic effect was also likely contributed by metoprolol. Existing literature has highlighted that the clinical response to beta-blockade therapy can be augmented by CYP2D6 polymorphisms. Hamadeh et al., in their meta-analysis, demonstrated an almost fourfold increased risk of bradycardia in patients with a reduced CYP2D6 clearance phenotype. The U.S. FDA label for ritonavir states that the drug has a moderate-to-strong CYP2D6 inhibitory effect, potentially converting a "normal" CYP2D6 phenotype into a poor metabolizer, increasing metoprolol serum concentrations and leading to further hemodynamic instability. Consequently, the FDA recommends caution to clinicians when prescribing ritonavir in patients taking beta-blockers and/or calcium channel blockers, and suggests that a dose reduction may be needed [7,11,12].

COVID-19 infection itself is known to potentiate bradycardia and other arrhythmias/heart blocks, making it challenging to distinguish between Paxlovid-mediated pathology versus viral manifestation in this case. The temporal relationship, however, of this patient’s hemodynamic collapse after administration of Paxlovid and subsequent resolution on discontinuation supports a CYP3A4/CYP2D6 inhibition effect as the likely culprit - simultaneously affecting levels of diltiazem (180 mg twice daily), ranolazine (1000 mg twice daily), and metoprolol tartrate (25 mg twice daily), supported by a Drug Interaction Probability Scale (DIPS) of 4 (possible) [13]. Furthermore, alternative etiologies, including metabolic derangements, thyroid dysfunction, myocardial ischemia, and inflammatory heart disease, were explored and ruled out. A noted limitation of this case, however, is the absence of measured serum drug levels during acute care for direct confirmation. Given the known CYP3A4 and CYP2D6 inhibition effects, the temporal series of events, and rapid clinical resolution following Paxlovid discontinuation, the conclusion remains inferential [8,13].

This case thus highlights the need for complete medication reconciliation by clinicians before opting to initiate Paxlovid antiviral therapy - particularly in patients with a complex multi-drug cardiovascular regimen. To mitigate life-threatening hemodynamic collapse from CYP metabolic insult, considerations should be made for alternative therapies or temporary dose reduction/suspension of cardiovascular medications, as recommended by the FDA.

## Conclusions

While COVID-19 can independently cause cardiac arrhythmias, the temporal relationship of nirmatrelvir/ritonavir (Paxlovid) initiation and the patient’s subsequent hemodynamic collapse, followed by rapid resolution upon drug discontinuation, strongly suggests a drug-drug interaction. The strong CYP 3A4 and moderate-to-strong CYP 2D6 inhibitor effect of ritonavir can lead to supratherapeutic levels of heart rate-suppressing medications, turning a standard outpatient treatment into a life-threatening bradycardic and hypotensive crisis. Hence, this case highlights the importance of a comprehensive medication reconciliation before prescribing Paxlovid. As recommended by the FDA, the dose reduction of high-risk cardiovascular therapies such as ranolazine, diltiazem, and metoprolol should be considered to avoid the risk of severe hemodynamic collapse.

## References

[1] Al-Rohaimi AH, Al Otaibi F (2020). Novel SARS-CoV-2 outbreak and COVID19 disease; a systemic review on the global pandemic. Genes Dis.

[2] Ahmad FB, Cisewski JA, Anderson RN (2022). Provisional mortality data - United States, 2021. MMWR Morb Mortal Wkly Rep.

[3] Ponnampalli S, Venkata Suryanarayana Birudukota N, Kamal A (2022). COVID-19: vaccines and therapeutics. Bioorg Med Chem Lett.

[4] Hsiao TK, Torvik VI (2023). OpCitance: citation contexts identified from the PubMed Central open access articles. Sci Data.

[5] Lam C, Patel P (2026). Nirmatrelvir-ritonavir. StatPearls [Internet].

[6] Joyce RP, Hu VW, Wang J (2022). The history, mechanism, and perspectives of nirmatrelvir (PF-07321332): an orally bioavailable main protease inhibitor used in combination with ritonavir to reduce COVID-19-related hospitalizations. Med Chem Res.

[7] (2026). U.S. Food and Drug Administration. Norvir (ritonavir) capsules, for oral use (prescribing information). Revised.

[8] Douedi S, Mararenko A, Alshami A (2021). COVID-19 induced bradyarrhythmia and relative bradycardia: an overview. J Arrhythm.

[9] Abraham S, Nooraie RY, Cheng-Lai A (2022). Cardiovascular drug interactions with nirmatrelvir/ritonavir in patients with COVID-19: JACC review topic of the week. J Am Coll Cardiol.

[10] Horn JR, Hansten PD (2016). CCBs and CYP3A4 inhibitors: watch out for enhanced cardiovascular response. Pharmacy Times.

[11] Duarte JD, Thomas CD, Lee CR (2024). Clinical Pharmacogenetics Implementation Consortium Guideline (CPIC) for CYP2D6, ADRB1, ADRB2, ADRA2C, GRK4, and GRK5 genotypes and beta-blocker therapy. Clin Pharmacol Ther.

[12] Meloche M, Khazaka M, Kassem I, Barhdadi A, Dubé MP, de Denus S (2020). CYP2D6 polymorphism and its impact on the clinical response to metoprolol: a systematic review and meta-analysis. Br J Clin Pharmacol.

[13] Horn JR, Hansten PD, Chan LN (2007). Proposal for a new tool to evaluate drug interaction cases. Ann Pharmacother.

